# Open Reduction of Subcondylar Fractures Using a New Retractor

**DOI:** 10.1155/2011/421245

**Published:** 2011-08-04

**Authors:** Akira Sugamata, Naoki Yoshizawa, Yoshio Jimbo

**Affiliations:** ^1^Department of Plastic and Reconstructive Surgery, Tokyo Medical University Hachioji Medical Center, 1163 Tatemachi, Hachioji, Tokyo 193-0998, Japan; ^2^Department of Plastic and Reconstructive Surgery, Kosei Hospital, 5-25-15 Yayoichou, Nakano, Tokyo 164-0013, Japan

## Abstract

Many operative approaches have been described for the open reduction of subcondylar fractures and rigid fixation. However, fracture portions are deep and embedded among facial nerves so that visual surgery in this region is extremely limited. Once the operative field is exposed, the displacement of the condylar head is often dislocated by the anteromedial pull of the lateral pterygoid muscle and the fracture end of the condylar process is pulled up to the mandibular fossa by contraction of the masseter muscle. We made a new retractor to achieve a better field of view. It is possible to pull down the condylar process by opening the tips of the retractor using the specially made wrench system without special effort and keep the condylar process in the same position during reduction. In using this retractor, the fracture stumps were clearly exposed and more easily reposited.

## 1. Introduction

Fracture around the condyle is the most common of all mandibular fractures [1, 2]. During surgery to repair such fractures, it is very important to ensure that the surgeon is able to conduct anatomic reduction and rigid internal fixation with direct vision of the dislocated condylar head. We have used the transparotid gland approach according to Jimbo et al. [3] and used our own retractor to pull down the condylar process and keep it in the same position during reduction. We introduce our method and the results of repairs to subcondylar fractures.

## 2. Retractor

**Figure 1:**
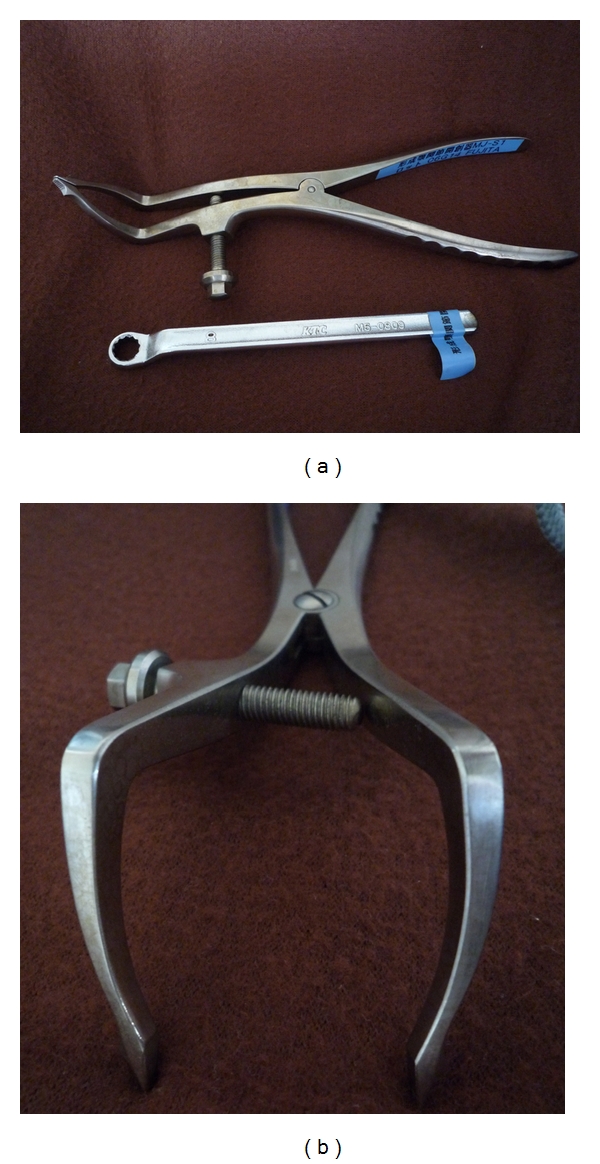
(a) The new mandibular joint retractor device, the length of which is 21 cm. (b) The top of the tip is 1 mm thickness and 3 mm width, the triangular projected portion is made 6 mm proximal from the top of the tip to catch fracture ends of the bone.

The retractor is 21 cm in length and made up of 10 cm tips and 11 cm handles. The tips are bent at one-third of the length from the distal point at an angle of 80°. The top of the tip is 1 mm in thickness and 3 mm in width; the triangular projected portion is made at 6 mm proximal to the top of the tip to catch the fracture ends of the bone. At 3.5 cm distal to the connected point of the left tip, the 5 mm diameter screw is attached. By turning this screw with the 12 cm length wrench, the tops of the tips are open gradually with high-grade opening force. The widest distance between the tips is 3 cm (Figure 1).

## 3. Operative Indication and Patients

Our current indication for open reduction of subcondylar fracture is complete dislocation of the condylar head from the mandibular fossa and the age of the patient being over twenty years old. For this displacement to occur, there must be rupture of the capsule, in which case the tip of the retractor can be inserted in the mandibular fossa.

From 2006 to 2010, 8 cases with subcondylar fractures were treated with the new retractor in our plastic surgery section. All patients were male, age range 17–56 (mean 34). Of the fractures among these patients, 2 were bilateral, 5 were on the left side, and one was on the right. The follow-up period ranged from 6 to 12 months (mean 10 months). Facial nerve function, degree of mouth opening, and occlusal relationship were assessed (Table 1).

## 4. Operative Method

**Figure 2:**
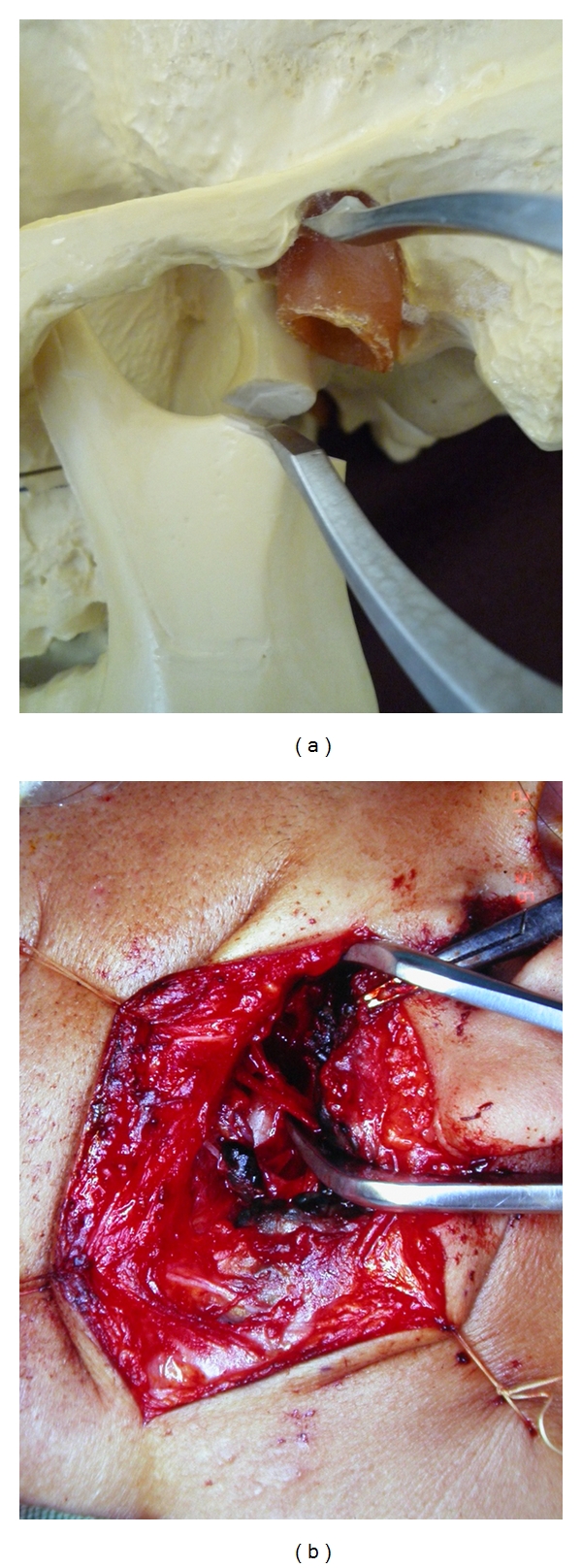
(a) It is possible to pull down the condylar process by opening the tips of the retractor. (b) Photograph shows the condylar head and the facial nerve between the tips of the retractor.

The surgical procedure required a 5–8 cm S-shape incision made from the ear lobe to the mandibular angle along the edge of the mandibular ramus. The subcutaneous tissue of the skin flap was raised forward to expose the anterior edge of the parotid gland. The fascia, which exists in the anterior portion of the parotid gland, was dissected vertically downward to expose the buccal and zygomatic branches of the facial nerve. The buccal and zygomatic branches of the facial nerve were exposed from the enveloped superficial lobe of the parotid gland in the direction of the main trunk. After the buccal and zygomatic branches were unfolded completely, the deep lobe of the parotid gland and the periosteum of the mandibular bone were dissected between the two branches to expose the fracture end of the mandibular condyle. Once the fracture end of the condylar process was exposed in the operative field, the tips of the new retractor device were inserted between the fracture end of the condylar process and the lateral margin of the mandibular fossa; the condylar process could then be pulled down by opening the tips of the retractor gradually using the specially made wrench system. Under this condition, the fracture stumps of the dislocated condylar head could be exposed (Figure 2). Then, using forceps, the condylar head could be pulled up between the two tips of the retractor. Simultaneous with the pulling up of the condylar head, the retractor was removed and anatomical reduction was performed. Once reduction of the condylar head was complete, one or two miniplates were set across the fracture line. The parotid gland and the parotid fascia were sutured firmly with 6–0 nylon thread. As the last step in the total procedure, IMF screws were inserted into the maxilla and the mandible. The day after the operation, loose intermaxillary fixation was set with elastic bands to more readily obtain a good occlusive relationship. One week after surgery, exercise of the mandibular joint was initiated under loose intermaxillary fixation to promote increased mobility of the mandibular joint, because long rigid intermaxillary fixation may cause ankylosis of the mandibular joint. One month after surgery, after the occlusion became stable, use of elastic bands was discontinued and IMF screws were removed to start mouth opening exercises.

## 5. Results

**Figure 3:**
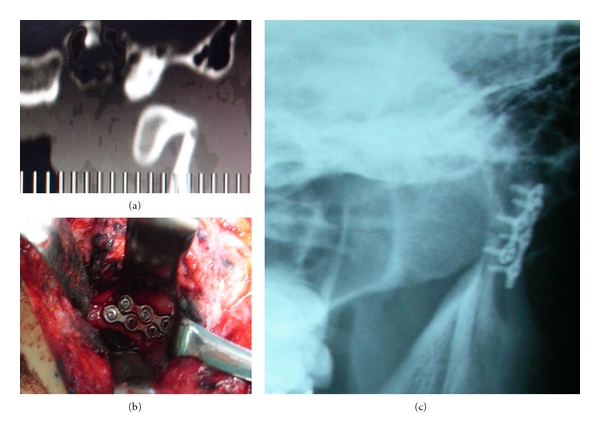
(a) CT shows a fracture of the left subcondyle with the condylar head dislocated anteromedially. (b) The condylar head was reposited, and two miniplates were fixed across the fractured line. (c) Postoperative radiography, showing reduction of the fracture segment.

**Figure 4:**
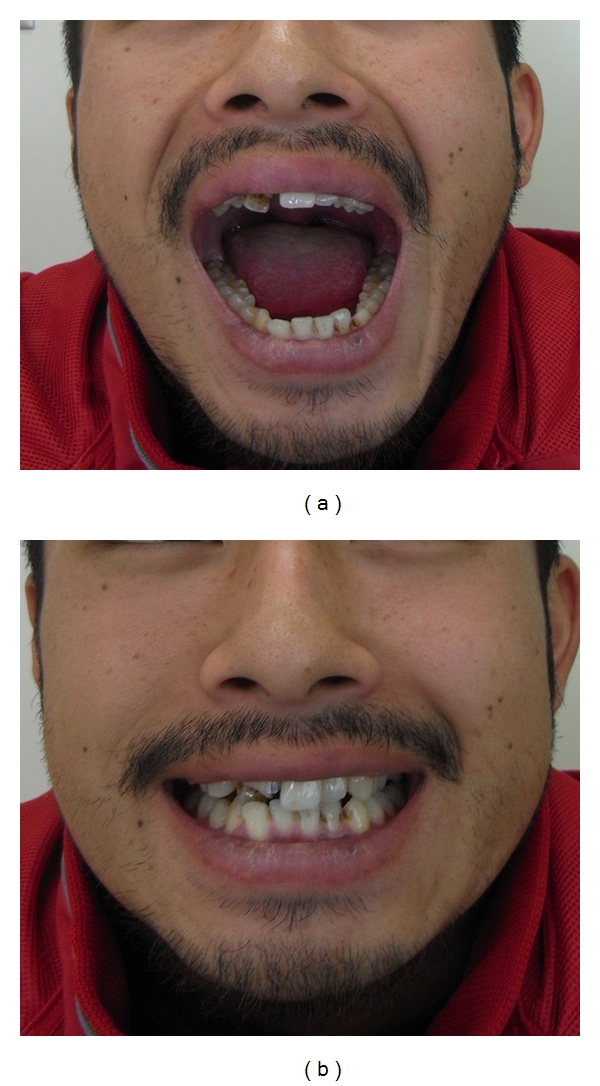
(a) Image shows good mouth opening with a slight deviation of the jaw (b) and good occlusion relationship.

Three months after surgery, a good occlusal relationship and satisfactory mouth opening were achieved in all patients (Figures 3 and 4). In two patients, the mandible leaned towards the affected side during wide opening of the mouth. One patient showed a slight weakness of the buccal branch of the facial nerve immediately after the operation; however, this resolved itself after two months.

## 6. Discussion

The reported incidence of mandibular condyle fractures ranges from approximately 30 to 50% of all mandibular fractures [1, 2]. The main controversies in condylar fractures relate to the basic philosophy of management. Both conservative and surgical treatment strategies have developed. However, if subcondylar fracture patients with dislocation of the condylar head from the mandibular fossa are treated conservatively, severe deviation of the jaw occurs frequently with opening. It is our recommendation that, in such cases, open reduction should be selected.

 Many operative approaches have been described for the open reduction and rigid fixation of subcondylar fractures [4–9]. However, fractures in this region are located beneath the parotid gland and facial nerves, such that visualization of the surgical field is extremely limited [4, 6]. In spreading out tissue to expose the operative field, the displacement of the proximal condylar head is often dislocated by the anteromedial pull of the lateral pterygoid muscle, and the proximal fracture end of the condylar process is pulled up to the mandibular fossa by contraction of the masseter muscle. To reposit the condylar head, it is necessary to pull down the condylar process from the mandibular fossa in a downward direction during surgery. Even if performed under muscle relaxant treatment, this procedure requires great effort. To keep the condylar process at the pulled down position during reduction, one assisting member of the surgical team is required to maintain a muscle retractor over a long time. Some types of retractors have been used previously to aid in fracture reduction [8]; however, these retractors are not useful for pulling down the condylar process because they do not produce a sufficiently high grade of opening force. Our new retractor device is very useful for pulling down the condylar process due to the high grade of the opening force of the tip of the retractor, which is achieved with the specially made wrench system. Furthermore, we can keep the condylar process in the pulled down position by maintaining the retractor with only one hand during reduction. By using this device, we can pull up the condylar head and conduct anatomic reduction more easily and under direct vision. 

Nevertheless, one must weigh the benefits against the potential complications that may be associated with the treatment of subcondylar fractures with this new device [10], even when the surgical outcomes are better. Because of the high-grade opening force, there may be some risk of additional fractures of the condylar process during the operation; however, we have not so far experienced such a complication in our patients. One patient showed temporary weakness of the buccal branch of the facial nerve, so it is recommended that careful handling of the device is required to avoid paralysis of the facial nerves due to compression or stretching of the nerves. However, there were no serious complications such as severe facial nerve paralysis associated with using the new retractor. Additionally, no patients showed severe ankylosis of the mandibular joint after the operation.

## 7. Conclusions

We have devised a new retractor to pull down the condylar process and obtain a better field of view during surgery. It is possible to produce a high-grade opening force at the tips of the retractor, which is achieved with the specially made wrench system. In using this retractor, the fracture stumps are more clearly exposed and easily repaired with surgery.

## Figures and Tables

**Table 1:** Patients' characteristics (*n* = 8).

Number	Sex	Age	Region	Occlusion and mouth opening	Complication
1	M	52	Bilateral	Good	(−)
2	M	45	Bilateral	Good	(−)
3	M	56	Left	Good	(−)
4	M	17	Left	Good	(−)
5	M	26	Right	Good	Temporary buccal
					Branch paralysis
6	M	21	Left	Good	(−)
7	M	32	Left	Good	(−)
8	M	19	Left	Good	(−)
